# Effect of *Theobroma cacao* L. on the Efficacy and Toxicity of Doxorubicin in Mice Bearing Ehrlich Ascites Carcinoma

**DOI:** 10.3390/antiox11061094

**Published:** 2022-05-31

**Authors:** Priyanka P. Patil, Pukar Khanal, Vishal S. Patil, Rajitha Charla, Darasaguppe R. Harish, Basanagouda M. Patil, Subarna Roy

**Affiliations:** 1 KLE College of Pharmacy Belagavi, KLE Academy of Higher Education and Research (KAHER), Belagavi 590010, Karnataka, India; priya.pp556@gmail.com (P.P.P.); pukarkhanal58@gmail.com (P.K.); vishalpatil6377@gmail.com (V.S.P.); 2 Indian Council of Medical Research, National Institute of Traditional Medicine, Belagavi 590010, Karnataka, India; rajitha.130484@gmail.com; 3 Department of Pharmacology, NGSM Institute of Pharmaceutical Sciences (NGSMIPS), Nitte (Deemed to be University), Mangalore 575018, Karnataka, India

**Keywords:** anticancer, cardiotoxicity, cocoa, doxorubicin, Ehrlich ascites carcinoma, *Theobroma cacao* L.

## Abstract

Background and objective: Doxorubicin is a widely used chemotherapeutic agent that causes oxidative stress leading to cardiotoxicity, hepatotoxicity, and nephrotoxicity. In contrast, *Theobroma cacao* L. has been recorded as an anticancer agent and found to be protective against multiple chemical-induced organ injuries, including heart, liver, and kidney injuries. The present study investigated the possible role of extracts from *T. cacao* beans for organ-protective effects in doxorubicin-induced toxicity in mice bearing Ehrlich ascites carcinoma (EAC). Methodology: After survival analysis in rodents, cocoa bean extract (COE) was investigated for its efficacy against EAC-induced carcinoma and its organ-protective effect against doxorubicin-treated mice with EAC-induced carcinoma. Results: Significant reductions in EAC and doxorubicin-induced alterations were observed in mice administered the COE, either alone or in combination with doxorubicin. Furthermore, COE treatment significantly increased the mouse survival time, life span percentage, and antioxidant defense system. It also significantly improved cardiac, hepatic, and renal function biomarkers and markers for oxidative stress, and it also reduced doxorubicin-induced histopathological changes. Conclusion: COE acted against doxorubicin-induced organ toxicity; potent antioxidant and anticancer activities were also reflected by the COE itself. The COE may therefore serve as an adjuvant nutraceutical in cancer chemotherapy.

## 1. Introduction

Doxorubicin, one of the well-established chemotherapeutic agents, is recommended in the management of breast, liver, kidney ovarian, thyroid cancer, Wilms’ tumor, and acute lymphoblastic and myeloblastic leukemia [1,2,3,4,5,6]. Doxorubicin exhibits its therapeutic activity by inhibiting the enzyme topoisomerase II and cleaving the DNA within tumor cells [7]. Despite its efficacy in managing the above-mentioned tumors and cancers, doxorubicin pharmacotherapy is limited due to its toxicity, mainly targeting vital organs, viz., the heart, liver, and kidney [8,9,10]. The suspected toxicity could be the outcome of the reactive oxygen species (ROS) generation and other Fenton reactions within the cytoplasm through the doxorubicin metabolite doxorubicinol [11,12,13,14].

Medicinal plants are being utilized by traditional healers to manage multiple communicable and non-communicable diseases due to the presence of various secondary bioactives, i.e., flavonoids, terpenes, alkaloids, and polyphenols [15,16]. Furthermore, due to the H-donating capacities of bioactives, extracts of various medicinal plants neutralize free radicals and terminate Fenton reactions, reducing oxidative stress in the cell [17]. Since doxorubicin pharmacotherapy is limited due to the free radical generation, it can be assumed that this limitation can be overcome with a secondary agent (medicinal plant with potent antioxidant properties) supplementation to neutralize the doxorubicin-generated free radicals. In addition, it may not only neutralize the doxorubicin-generated free radicals but may also uplift the effectiveness of doxorubicin chemotherapy if it possesses anticancer properties.

*Theobroma cacao* L., commonly known as cocoa, belongs to the family Sterculiaceae, which is native to Central America. Its nibs were reported to possess multiple pharmacological spectra due to the presence of various secondary metabolites, i.e., flavonoids, polyphenols, and alkaloids [18]. Furthermore, the efficacy of cocoa as a free radical scavenger [19], anti-inflammatory substance [20], and anticancer agent [21,22], and exhibiting cardioprotective [23], nephroprotective [24], and hepatoprotective activities [25], have been reported. Treatment with extracts of cocoa leaves and bark have shown protection against doxorubicin-induced oxidative stress and organ damage (hepatotoxicity, nephrotoxicity, and splenotoxicity) in rats [26,27]. In addition, cocoa extract has been reported for its cytotoxic activity against cancer cell lines [21,28]. Antioxidants present in cocoa possess free radical scavenging properties and can reduce free radical formation. Although cocoa has been demonstrated to ameliorate doxorubicin-mediated organ toxicity in non-carcinogen animals, its effect on EAC-induced carcinoma has not yet been reported. Hence, we hypothesized that cocoa extract supplementation with doxorubicin could ameliorate organ toxicity and enhance life expectancy. Thus, based on the above hypothesis, the present study was carried out to investigate the effect of hydroalcoholic extract of cocoa bean (COE) supplementation on doxorubicin-induced cardiotoxicity, hepatotoxicity, and nephrotoxicity in EAC mice and to assess its anticancer efficacy, if any, in combination with doxorubicin treatment.

## 2. Materials and Methods

### 2.1. Plant Collection, Authentication, and Preparation of Extract

Cocoa pods were collected from Sirsi (14°0.34′38.7984″ N, 74°0.58′21.288″ S) n the Uttar Kannada District of Karnataka, India, authenticated by a taxonomist at ICMR-NITM Belagavi, and the herbarium (voucher number: RMRC-1392) was deposited for future reference.

For extraction, the collected pods were thoroughly washed under running water, chopped, shade dried, and turned into coarse power using a pulverizer, defatted using petroleum ether (bp 40–60 °C), subjected to maceration (80% ethanol) in a closed container for a week and then filtered, concentrated, lyophilized, and stored in an airtight container for further use [29].

### 2.2. Extract Yield and Preliminary Phytochemical Analysis

After successful extraction, the percentage yield per 100 g of coarse powder was calculated for COE, followed by the total phenol and total flavonoid content estimation.

#### 2.2.1. Total Phenol Content

The total phenol content of the extract was estimated using the modified Folin–Ciocalteu method [30]. Briefly, the COE/gallic acid was mixed with 2 mL of Folin–Ciocalteu reagent (diluted with 1:10 *v*/*v* water) and 2 mL (75 g/L) sodium carbonate. The tubes were stirred at 1000× *g* for 15 s and allowed to stand at 25 °C for 20 min to produce colour. The absorbance at 760 nm was then measured using a UV spectrophotometer (Shimadzu (UV-1601) Kyoto, Japan). The total phenol content was expressed in terms of micrograms gallic acid equivalents (GAE) per milligram of extract.

#### 2.2.2. Total Flavonoid Content

Total flavonoid content was calculated using Zhisten’s method [31], where 100 µg/mL of COE was prepared and dissolved in ethanol. Then, 1 mL of COE was added to a test tube with 4 mL of distilled water. During the zero points, 0.3 mL of 5% sodium nitrite was added. After 5 min, 0.3 mL of 10% aluminum chloride was added. After 6 min, 2 mL of 1 M sodium hydroxide was added. Immediately, the mixtures were diluted with 2.4 mL of distilled water and mixed well. The absorption of the developed pink colour was reported at 510 nm, relative to the blank value. The standard curve for total flavonoids was plotted using quercetin solution (20 to 100 µg/mL) under the same procedure as previously described. The total flavonoid content was expressed in milligrams of quercetin equivalent (QCE) per milligram of extract.

### 2.3. Animal Studies

#### Ethical Clearance and Animal Procurement

The study protocol was reviewed and approved by the Institutional Animal Ethical Committee of ICMR-NITM, Belagavi (accession number: IAEC/ICMR-NITM BGM/2018/3) for experimentation with animals. Healthy Balb/c female mice (22–25 g) were procured from M/s in vivo Biosciences Bangalore (Karnataka, India) and maintained under controlled conditions of temperature (23 ± 2 °C), humidity (50 ± 5%), and light (12/12 h of light and dark, respectively) at the Animal Research Facility, ICMR-NITM, for experimentation.

### 2.4. Induction of Tumors

Mice carrying Ehrlich ascites carcinoma (EAC) tumor cells were obtained from M/s In vivo Biosciences Bangalore (Karnataka, India), and these cells were maintained and propagated by serial intraperitoneal transplantation of 1 × 10^6^ cells into healthy mice in an aseptic environment. The cells were propagated for 14 days and were used in the experiments.

### 2.5. Survival Time in EAC Mice

A total of 50 female adult Balb/c mice (22–25 g) were randomized using computer-generated random numbers into 5 groups (n = 10), in which animals were injected with EAC except one group that served as normal and received vehicle (2% gum acacia). Grouping was as: (1) Normal: received 2% gum acacia p.o.; (2) EAC: received EAC + 2% gum acacia p.o.; (3) DOX: received doxorubicin (4.91 mg/kg, i.p., q.wk. calculated based on the therapeutic equivalent dose of doxorubicin [32]; (4) COE: received COE 200 mg/kg, p.o; COE dose was selected based on acute toxicity studies and pilot safety studies; (5) COE + DOX: received (COE 200 mg/kg, p.o. o.d. + doxorubicin 4.91 mg/kg, i.p., q.wk.) for 28 days and observed daily for survival analysis. The mean survival time [33] was caculated as

$$MST=\frac{\Sigma \;Survival\;time\;\left(days\right)\;of\;each\;mouse\;in\;a\;group}{Total\;number\;of\;mice}$$

$$Increased\;life\;span\;\left(\%\right)=\frac{MST\;of\;treated\;mice}{MST\;of\;the\;cancer\;control\;group}\times 100\;$$

### 2.6. Evaluation of the Anticancer, Cardioprotective, Hepatoprotective, and Nephroprotective Activities of COE Alone and in Combination with Doxorubicin in the EAC Tumor Model

Based on the outcome of the above study, we designed the experiment for 21 days of treatment after tumor inoculation/induction. To study the prophylactic activity of COE on cancer progression and doxorubicin-induced toxicities, we included a COE-pretreated group (Pt). In this group, animals received COE for 21 days before tumor cell inoculation, and the rest of the treatment was the same as in group 5 (COE + DOX).

#### Animal Grouping and Experimentation

Sixty female adult Balb/c mice (22–25 g) were randomized using computer-generated random numbers into 6 groups (n = 10), in which animals were inoculated (i.p.) with EAC to induce tumors, except the normal group. Grouping was as (1) Normal: received 2% gum acacia p.o.; (2) EAC: received EAC + 2% gum acacia p.o.; (3) DOX: received doxorubicin (4.91 mg/kg, i.p., q.wk.); (4) COE: received COE 200 mg/kg, p.o.; (5) COE + DOX: received (COE 200 mg/kg, p.o. + doxorubicin 4.91 mg/kg, i.p. q.wk.); (6) Pt + COE + DOX: normal animals pretreated with COE (200 mg/kg, p.o.) for the previous 21 days, EAC was induced on the 23rd day after receiving (COE 200 mg/kg, p.o. + doxorubicin 4.91 mg/kg, i.p., q.wk.) for another 21 days.

During treatment, the body weight of each animal was measured at an interval of 3 days until the end of the study. On the 22nd day, the electrocardiogram (ECG) of individual animals from each group was recorded under anesthesia with diazepam (3 mg/kg) [34]. After successful completion of the ECG record, blood was drawn into 2 tubes, one in an EDTA tube for CBC and another in a microcentrifuge tube for serum separation and estimation of various parameters. Animals were finally euthanized by ketamine overdose, and the tumor volume and weight of the vital organs (heart, liver, and kidney) were recorded. Furthermore, portions of each heart, liver, and kidney tissue were homogenized and used for the antioxidant assay. The remaining tissue was fixed in a 10% formalin solution for histopathological studies.

- Electrocardiogram

Myocardial injury electrocardiograph (ECG) recordings were considered for the diagnosis of cardiac abnormalities in doxorubicin-treated mice at the end of the study. Mice were anaesthetized with diazepam (3 mg/kg) [34], and ECG was carried out. The electrocardiograms were recorded using the MP35, Biopac 4.0 system. Briefly, after anesthesia, the mouse was fixed on a foam plate, ECG patches were placed on the mouse’s leg and arm, connected with electrodes, and the two leads were used to collect signals. The red electrode was connected to the upper left limb, the black electrode was connected to the lower left limb, and the white electrode was connected to the right forelimb. ECG was recorded for at least 60 s. The ECG recording speed was 30 mm/s and the voltage was 1 mV/cm. ECG wave analysis was performed to calculate heart rate (beats/minute) and QRS complex (ms) using the software.

b. Evaluation of EAC Volume and Percentage Change in Body Weight

The change in body weight (BW) was recorded at intervals of three days until the completion of an experiment. After 24 h from the last treatment, the mice were sacrificed, and ascitic fluid was aspirated from the mice to estimate the EAC volume.

c. Serum Biochemical Parameters

At the end of the experiment, blood samples were taken from the medial canthus of the eye of the mice in all groups. Blood was collected in two tubes, one in the EDTA tube for haematological evaluation and another for separation of the serum for biochemical analysis of heart, liver, and kidney function. Cardiac biomarkers creatinine kinase-MB (CPK-MB) and lactate dehydrogenase (LDH); serum lipid profiles, such as triglycerides, total cholesterol, low-density lipoproteins (LDL), and high-density lipoproteins (HDL); the liver markers serum glutamic-oxaloacetic transaminase (SGOT), serum glutamic pyruvic transaminase (SGPT), and alkaline phosphatase (ALP); and serum creatinine and blood urea nitrogen for kidney markers were quantified using commercially available kits (Biosystems, S.A., Barcelona, Spain) in a semi-auto analyser (A15 Biosystems S.A., Barcelona, Spain).

d. Haematological Evaluations

Complete blood counts (red blood cells (RBC), White blood cells (WBC), platelets, mean corpuscular volume (MCV), mean corpuscular haemoglobin concentration (MCHC), % Hb, mean corpuscular haemoglobin (MCH), mean cell haemoglobin concentration and packed cell volume) were measured using an automatic haematology analyser (Erba H560).

e. Quantification of Enzymatic and Non-Enzymatic Antioxidant Biomarkers in the Tissue Homogenate (Heart, Liver, and Kidney)

Mouse heart, liver, and kidney homogenates were tested to quantify multiple enzymatic and nonenzymatic antioxidant parameters. Organs were dissected out and washed in cooled saline, blotted on filter paper, weighed, and homogenized in cold phosphate buffer (0.05 M, pH 7.4). The homogenates were centrifuged at 10,000 rpm for 10 min at 4 °C, and post-mitochondrial supernatant (PMS) was used to assess total protein and lipid peroxidation. The supernatant was again centrifuged at 15,000 rpm at 4 °C for 1 h. The supernatant obtained was used to estimate superoxide dismutase (SOD) [35], catalase (CAT) [36], glutathione (GSH) [37], lipid peroxidation (LPO) [38] and total thiols [37].

f. Histopathological Investigations of the Heart, Liver, and Kidney

Three samples of heart, liver, and kidney tissues from each group of mice were fixed in 10% formalin solution for histopathological studies. The tissue slices were examined at 40× magnification using eosin haematoxylin dye.

### 2.7. Statistical Analysis

Data are expressed as the mean ± SEM. The difference between the means between the groups was analysed using one-way ANOVA followed by a post hoc Tukey’s test using GraphPad Prism version 5. The difference between the means was considered to be significant if *p* < 0.05.

## 3. Results

### 3.1. Extraction Yield and Preliminary Phytochemical Analysis

The yield of COE was 5.8% *w*/*w* per 100 g dried powder. Preliminary phytochemical analysis revealed the presence of phenols and flavonoids in COE. The total phenolic content of the extracts estimated using the calibration curve’s regression equation (y = 0.0192x + 0.0257; R^2^ = 0.992) reported in mg gallic acid equivalents (GAE) was 72.5 µg GAE/mg of extract. The total flavonoid content of the COE was determined using the calibration curve’s regression equation (y = 0.0009x + 0.0237, R^2^ = 0.9801) reported in mg quercetin equivalents (QE) was 165 µg QE/mg of extract.

### 3.2. General Observation and Survival Time

Mice in the EAC group showed loss of locomotor activity, reduced appetite, swollen abdomen, and a vicious bloody ascitic fluid compared with mice in other intervention groups. However, the improvement was high in the COE group, moderate in the COE + DOX group and low in the DOX group. It was also observed that DOX group animals developed scruffy fur, red exudate around the eyes, soft watery feces, and signs of necrosis at the site of doxorubicin injection.

To study the effect of COE and COE + DOX on improving survival time, the treatment was provided, and mice were observed throughout the experiment (60 days) with a pellet diet and ad libitum access to water. No mortality was observed in the normal group throughout the study. However, in EAC-bearing animals, the mean survival time (MST) was 21 days. In the doxorubicin-treated group, the MST was 25 days, and the percentage increased life span (ILS) was 19.04%. Similarly, in the COE group, the MST extended to 33 days, and the % ILS was found to be 57.14%. In the COE + DOX group of animals, the MST was 28 days, and the % ILS was found to be 33.33% compared with the EAC alone groups.

**Figure 1:**
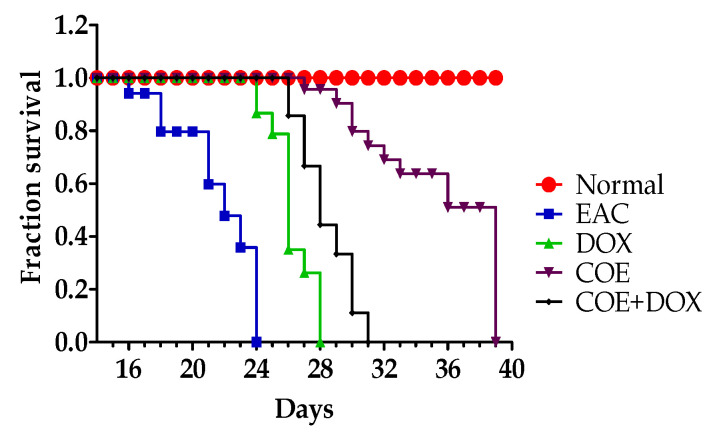
Effect of COE on survival time in EAC mice.

There was a significant difference (*p* = 0.0003; *p* < 0.001) in the Mantel–Cox log-rank between the groups with 21.31 χ^2^; also, the Gehan–Breslow–Wilcoxon test reflected a significant difference (*p* = 0.0019; *p* < 0.01) between the groups with 17.01 χ^2^. Figure 1 represents the survival time in EAC mice.

### 3.3. Electrocardiogram

**Figure 2:**
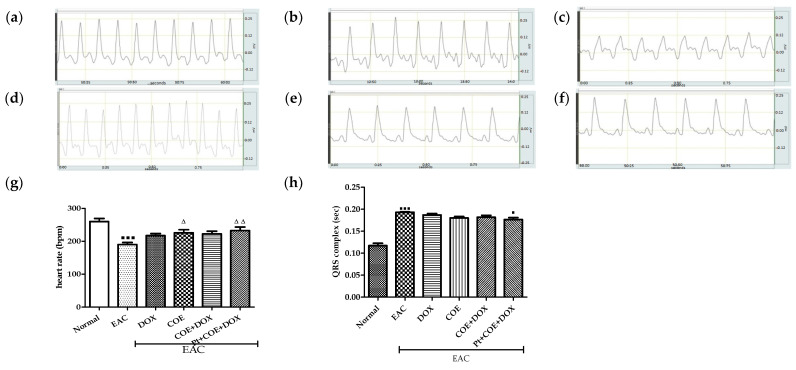
Effect of COE on ECG on doxorubicin-induced cardiomyopathy in EAC-induced carcinoma. Qualitative analysis of ECG of all the experimental groups; (**a**) Normal, (**b**) EAC, (**c**) DOX, (**d**) COE, (**e**) COE + DOX, (**f**) Pt + COE + DOX, (**g**) effect on heart rate, (**h**) effect on QRS complex. All values are expressed as the mean ± SEM (n = 6). Data were analysed using a one-way analysis of variance (ANOVA) followed by Tukey’s test. ^▀^ *p* < 0.05, ^▀^^ ^^▀^^ ^^▀^ *p* < 0.001, compared with normal; ^Δ^ *p* < 0.05, ^ΔΔ^ *p* < 0.01, compared with EAC.

Mice in the EAC group showed a significant decrease (*p* < 0.001) in heart rate compared with the normal group, which was significantly reversed in the COE (*p* < 0.05) and pretreated groups (*p* < 0.01) when compared with EAC. Furthermore, there was a significant increase (*p* < 0.001) in the QRS complex in the EAC group compared with the normal group. No significant change was observed in the QRS complex in any of the groups except the Pt + COE + DOX group (*p* < 0.05) compared with the EAC group. However, an observable decrease in the amplitude of the QRS complex was recorded in the COE-treated group compared with the EAC and DOX groups (Figure 2).

### 3.4. Physical Parameters, Ascitic Fluid Volume, and % Change in Body Weight

**Figure 3:**
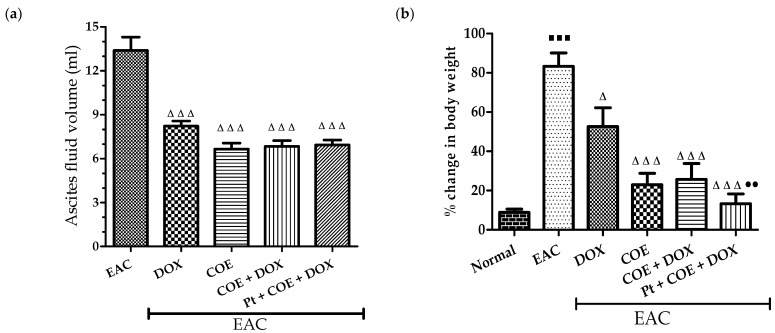
Effect of COE on ascitic fluid volume (**a**) and % change in body weight (**b**). All values are expressed as the mean ± SEM (n = 6). Data were analysed using one-way analysis of variance (ANOVA) followed by Tukey’s test. ^▀^^ ^^▀^^ ^^▀^ *p* < 0.001, compared with normal; ^Δ^ *p* < 0.05, ^Δ^ ^Δ^ ^Δ^ *p* < 0.001, compared with EAC; ^●●^ *p* < 0.01 compared with DOX.

A significant decrease (*p* < 0.001) in the ascitic fluid volume was observed in the COE and COE + DOX groups compared with the EAC group. Furthermore, the percentage change in body weight was observed to be significantly higher (*p* < 0.001) in the EAC group than in the normal group, which was significantly reversed in the COE (*p* < 0.001), DOX (*p* < 0.05), COE + DOX (*p* < 0.001) and pretreatment (*p* < 0.001) groups compared with the EAC group. Additionally, COE pretreatment reflected a significant decrease (*p* < 0.01) in the % change in body weight compared with the doxorubicin alone treatment group (Figure 3).

### 3.5. Organ Weight: Heart, Liver, and Kidney

In the EAC group, a significant decrease (*p* < 0.001, 0.05) in the heart weight was observed compared with the normal group. However, no significant difference in heart weight was observed if compared with the treatment groups. In contrast to the heart weight, there was a significant increase in the liver (*p* < 0.001) and kidney (*p* < 0.05) weights in the EAC group compared with the normal group. Liver weights were significantly reduced in all the treatment groups compared with the EAC group. Furthermore, there was a significant increase (*p* < 0.05) in kidney weight in the EAC group compared with the normal group, but none of the intervention groups showed a significant weight reduction when compared with the normal group (Table 1).

### 3.6. Haematology

There was a significant decrease (*p* < 0.001) in blood RBC, Hb, and PCV, while an observable reduction in platelet count, lymphocytes, and MCH levels was observed in the EAC group compared with the normal group. The same blood parameters remained significantly improved (*p* < 0.05, 0.01, 0.001, respectively) in animals administered COE or doxorubicin, either alone or in combination. In contrast, there was a significant increase (*p* < 0.001) in MCHC and WBC, and an observable increase in eosinophils and monocytes in the EAC group compared with the normal group. In addition, the levels of these blood markers were significantly altered (*p* < 0.05, 0.01, 0.001, respectively) towards the normal value in COE and DOX-treated animals as well as in combination. Furthermore, maximum improvement in ameliorating the haematological parameters occurred in COE-pretreated mice (Table 2).

### 3.7. Cardiac CPK-MB and LDH Levels

A significant increase in cardiac CPK-MB (*p* < 0.05) levels was observed in the EAC group compared with the normal group. The DOX group showed an increase (nonsignificant) in CPK-MB levels compared with the EAC group. Furthermore, the COE-treated group showed a significant decrease (*p* < 0.05) in CPK-MB levels, whereas the COE + DOX and Pt + COE + DOX groups showed an increase (non significant) in CPK-MB levels compared with the EAC group. A significant increase (*p* < 0.001) in LDH levels was observed in the EAC group compared with the normal group. Furthermore, doxorubicin treatment showed a significant increase (*p* < 0.001) in LDH levels compared with the EAC group. Treatment with COE, COE + DOX, and Pt + COE + DOX caused a significant decrease (*p* < 0.001) in LDH levels compared with the EAC group. The LDH levels in the COE, COE + DOX, and Pt + COE + DOX treated groups were found near to the normal group. Notably, the LDH level was significantly reduced (*p* < 0.001) in the COE + DOX and pretreatment groups compared with the DOX group. Additionally, it was observed that prophylactic and curative treatment with COE alone or in COE + DOX resulted in a significant amelioration (*p* < 0.001) of cardiac marker levels (Table 3).

### 3.8. Hepatic Enzymes

Significant increases (*p* < 0.001) in ALP, SGOT, and SGPT levels were observed in the EAC group compared with the normal group. In addition, the DOX group showed a significant decrease in ALP (*p* < 0.001), SGOT (*p* < 0.05), and SGPT (*p* < 0.001) compared with the EAC group. In contrast, the COE + DOX and Pt + COE + DOX groups showed significant decreases (*p* < 0.001) in ALP, SGOT, and SGPT levels compared with the EAC group, whereas only SGOT and SGPT were observed to decrease compared with the DOX group. Similarly, the COE group also showed a significant decrease (*p* < 0.001) in ALP, SGOT, and SGPT levels compared with the EAC group (Table 4).

### 3.9. Kidney Markers

Significant increases (*p* < 0.001) in creatinine and blood urea nitrogen (BUN) levels were observed in the EAC group compared with the normal group. However, these levels were significantly reversed (*p* < 0.001) in all the treatment groups. Additionally, there was a significant decrease in BUN levels in the COE + DOX group (*p* < 0.05) and COE pretreated group (*p* < 0.001) compared with the DOX group. However, there was an observable difference in creatinine levels in the COE + DOX and Pt + COE + DOX groups compared with the DOX group (Table 5).

### 3.10. Lipid Profile

A significant decrease (*p* < 0.001) in HDL levels was observed in the EAC group compared tonormal group. The doxorubicin-treated group also showed a decrease in HDL levels compared with the EAC group. However, HDL levels were improved in the COE, COE + DOX, and Pt + COE + DOX groups. Furthermore, LDL levels significantly decreased in the DOX (*p* < 0.01), COE (*p* < 0.05), and COE + DOX (*p* < 0.05) groups. In addition, cholesterol levels significantly increased (*p* < 0.01) in the DOX group and significantly decreased (*p* < 0.001) in the COE group compared with the EAC group (Table 6).

### 3.11. Enzymatic and Nonenzymatic Antioxidant Biomarkers in Heart Homogenate

A significant increase in LPO (*p* < 0.001) levels was observed along with a significant decrease in GSH (*p* < 0.001), SOD (*p* < 0.001), CAT (*p* < 0.001), and total thiols (*p* < 0.01) in the EAC group compared with the normal group. The levels of these markers were significantly reversed (*p* < 0.01 to *p* < 0.001) in all the treatment groups compared with the EAC group. However, in the DOX group, the GSH level was found to be nonsignificant varying from that in the EAC group. Interestingly, in the COE + DOX and Pt + COE + DOX groups a significant decrease in LPO (*p* < 0.05, *p* < 0.05, respectively) and a significant increase in SOD (*p* < 0.01, 0.05, respectively) and total thiol (ns, *p* < 0.05, respectively) levels was observed, compared with the DOX group. Furthermore, there was an increase in GSH levels in the COE + DOX group and Pt + COE + DOX group (*p* < 0.05) compared with the DOX group (Table 7).

### 3.12. Enzymatic and Nonenzymatic Antioxidant Biomarkers in Hepatic Homogenate

Significant increases (*p* < 0.001) in LPO levels and significant decreases in GSH (*p* < 0.001), SOD (*p* < 0.001), CAT (*p* < 0.001), and total thiol (*p* < 0.01) levels were observed in the EAC group compared with the normal group. The levels of these markers were significantly (*p* < 0.001) improved in the COE, COE + DOX, and Pt + COE + DOX groups compared with the EAC group. A significant decrease in LPO levels (*p* < 0.001) and increases in GSH (ns), SOD (*p* < 0.001), CAT (*p* < 0.001), and total thiol (*p* < 0.05) levels were observed in the DOX group compared with the EAC group. Furthermore, in the COE + DOX and Pt + COE + DOX groups, there were significant decreases in LPO (ns, *p* < 0.05, respectively) and increases in SOD (ns, *p* < 0.05, respectively) and total thiol (*p* < 0.001, *p* < 0.01, respectively) levels, compared with the DOX group (Table 8).

### 3.13. Enzymatic and Nonenzymatic Antioxidant Biomarkers in Kidney Homogenate

A significant increase (*p* < 0.001) in the LPO level and a significant decrease in GSH (*p* < 0.001), SOD (*p* < 0.001), CAT (*p* < 0.001), and total thiols (*p* < 0.001) was observed in the EAC group in comparison with the normal group. A significant reduction in the LPO level (*p* < 0.001) was observed in all treatment groups compared with the EAC group. Furthermore, in the COE, COE + DOX, and Pt + COE + DOX groups, a significant increase in GSH (*p* < 0.001, 0.001, 0.001, respectively), SOD (*p* < 0.001, 0.05, 0.01, respectively), CAT (*p* < 0.001, 0.05, 0.05, respectively), and total thiols (*p* < 0.01, ns, 0.05, respectively), was observed compared with the EAC group. Furthermore, it was observed that there was a significant increase (*p* < 0.001) in GSH levels in the COE + DOX and Pt + COE + DOX groups when compared with the DOX group (Table 9).

### 3.14. Histopathology

#### 3.14.1. Histopathology of the Heart

**Figure 4:**
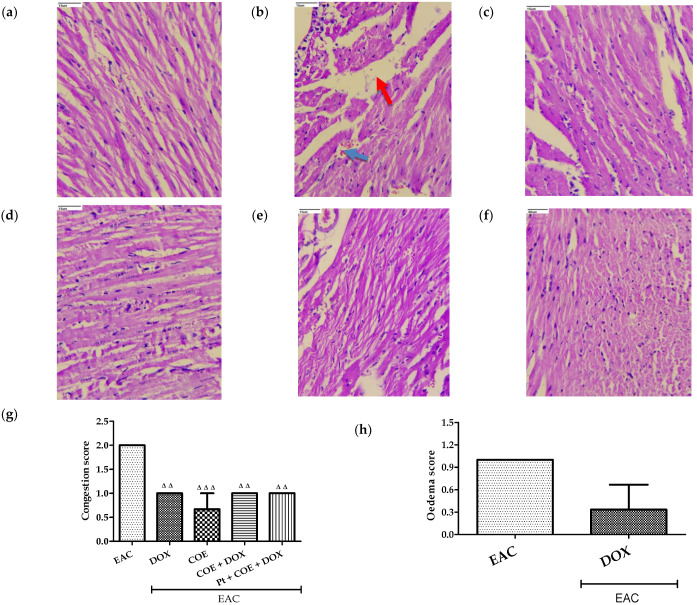
Effect of COE on cardiac histology. Photograph of heart sections of different treatment groups stained with haematoxylin and eosin. Plates at 40× magnification. (**a**) Normal, (**b**) EAC, (**c**) DOX, (**d**) COE, (**e**) COE + DOX, (**f**) Pt + COE + DOX, (**g**) Congestion score, and (**h**) Oedema score. EAC group (**b**) showing oedema (red arrow) and congestion (blue arrow). All values are expressed as the mean ± SEM (n = 3). Data were analysed using one-way analysis of variance (ANOVA) followed by Tukey’s test ^ΔΔ^ *p* < 0.01, ^ΔΔΔ^ *p* < 0.001 compared with EAC.

The H&E staining of cardiac tissue in the normal group of animals revealed normal morphology of myocardial cells with complete and orderly myocardial fibres and normal myocardial interstitial (Figure 4a). However, myocardial cells in the animals of the EAC and DOX groups showed inflammation, a widened intermuscular plane, and broken and disorganized myocardial fibres (Figure 4b,c). However, these features were attenuated in the COE, COE + DOX, and pretreatment groups compared with the EAC and DOX groups (Figure 4d–f). Similarly, congestion was found to be significantly decreased in all treated groups compared with the EAC group (Figure 4g). Edema was observed only in the EAC and DOX alone treatment groups. These results suggest that COE administration alone or in COE + DOX ameliorates cardiac remodeling. The bar graph shows the scoring of the pathological changes (Figure 4).

#### 3.14.2. Histopathology of Liver

**Figure 5:**
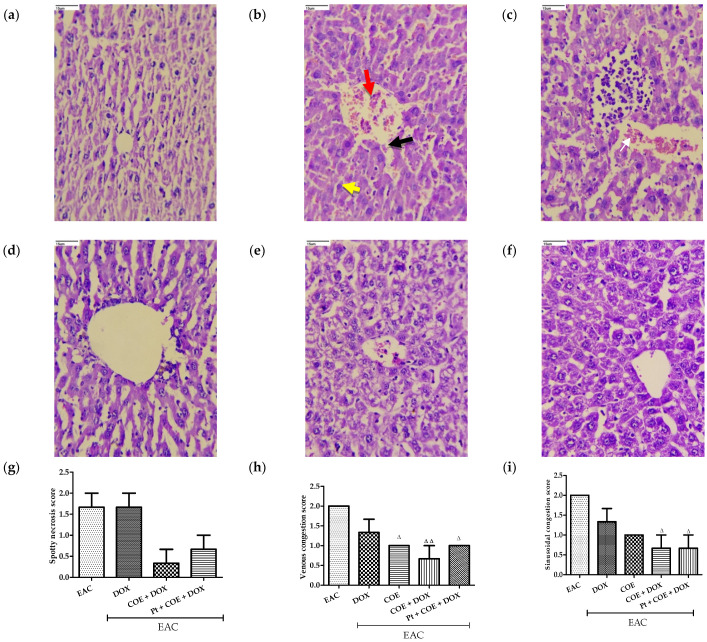

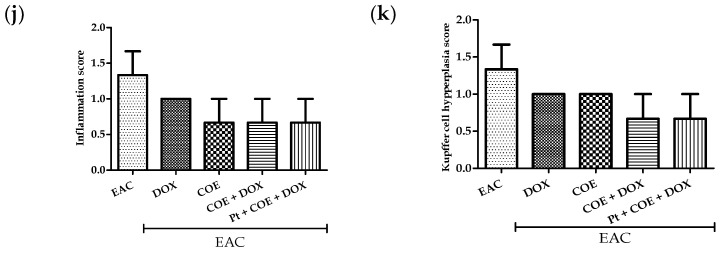
Effect of COE on liver histology. Photograph of liver sections of different treatment groups stained with haematoxylin and eosin. Plates at 40× magnification. (**a**) Normal, (**b**) EAC, (**c**) DOX, (**d**) COE, (**e**) COE + DOX, (**f**) Pt + COE + DOX, (**g**) Spotty necrosis score, (**h**) Venous congestion score, (**i**) Sinusoidal congestion score, (**j**) Inflammation score, (**k**) Kupffer cell hyperplasia score. EAC group (**b**) showing central vein congestion (red arrow), venous congestion (black arrow), inflammation (yellow arrow) and DOX group (**c**) showing sinusoidal congestion (white arrow). All values are expressed as the mean ± SEM (n = 3). Data were analysed using one-way analysis of variance (ANOVA) followed by Tukey’s test. ^Δ^ *p* < 0.05, ^ΔΔ^ *p* < 0.01, compared with EAC.

The H&E-stained liver sections of normal mice showed typical hepatic tissue architectures. The EAC group and DOX alone group showed spotty necrosis, inflammation, and venous congestion areas in hepatic cells (Figure 5b,c). However, the COE, COE + DOX, and Pt + COE + DOX groups showed improved regeneration of hepatic tissue with a lesser degree of inflammation, sinusoidal congestion, venous congestion, and Kupffer cell hyperplasia in the hepatic section (Figure 5d–f). No spotty necrosis was observed in the COE–treated animals, and a noticeable decrease in spotty necrosis was found in animals in the COE + DOX and Pt + COE + DOX groups. Furthermore, there was a significant decrease in venous congestion in the COE and Pt + COE + DOX groups (*p* < 0.05) and in the COE + DOX group (*p* < 0.01) compared with the EAC group. No significant improvement was found in the DOX alone group. Similarly, sinusoidal congestion was significantly ameliorated in the COE + DOX and Pt + COE + DOX (*p* < 0.05) groups. The bar graph shows the scoring of the pathological changes (Figure 5).

#### 3.14.3. Histopathology of Kidney

**Figure 6:**
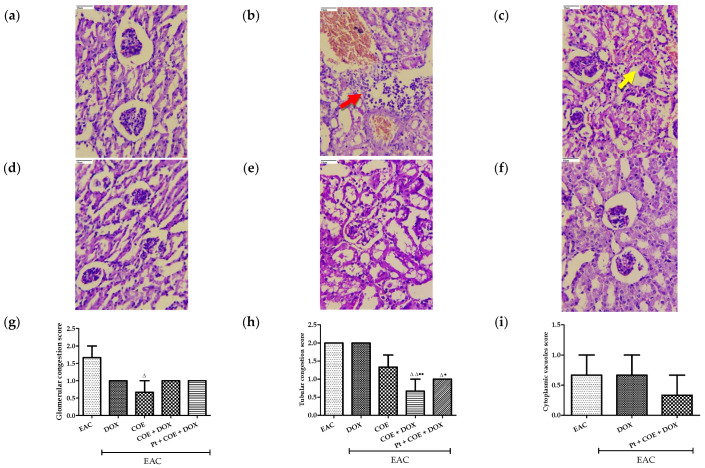

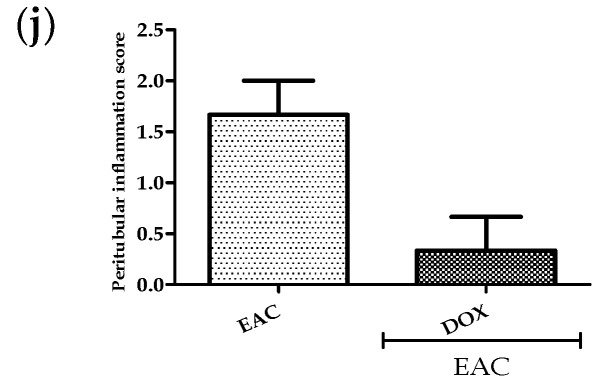
Effect of COE on kidney histology. Photograph of kidney sections of different treatment groups stained with haematoxylin and eosin. Plates at 40× magnification. (**a**) Normal, (**b**) EAC, (**c**) DOX, (**d**) COE, (**e**) COE + DOX, (**f**) Pt +COE + DOX, (**g**) Glomerular congestion score, (**h**) Tubular congestion, (**i**) Cytoplasmic vacuoles score, (**j**) Peritubular inflammation score. EAC group (**b**) showing peritubular inflammation (red arrow) and DOX group (**c**) showing tubular congestion (yellow arrow). All values are expressed as the mean ± SEM (n = 3). Data were analysed using one-way analysis of variance (ANOVA) followed by Tukey’s test. ^Δ^ *p* < 0.05, ^ΔΔ^ *p* < 0.01, compared with EAC; ^●^ *p* < 0.05, ^●●^ *p* < 0.01, compared with DOX.

The histopathological examination of kidney tissue showed an observable decrease in glomerular congestion, tubular congestion, cytoplasmic vacuoles, and peritubular inflammation within all treatment groups compared with animals in the EAC group. The bar graph shows the scoring of the pathological changes in Figure 6.

## 4. Discussion

In our present study, we demonstrated the effects of COE on the heart, liver, and kidney in doxorubicin-treated EAC-bearing mice. Treatment with COE was found to negate the adverse effects of doxorubicin on these organs, as evidenced by significant improvement of the above-mentioned organs’ biomarkers in animals treated with COE in combination with doxorubicin. COE not only reduced the organ toxicity associated with doxorubicin but also acted as an anticancer agent and promoted the anticancer activity of doxorubicin. Treatment with COE alone and in combination with doxorubicin in tumor-bearing mice showed a significant deceleration of cancer progression compared with doxorubicin alone treatment. Additionally, COE alone and in combination with doxorubicin resulted in a significant increase in the MST (33 and 28 days, respectively) compared with doxorubicin alone treatment (25 days) and the EAC group (21 days). These results indicate that COE did not interfere with the anticancer activity of doxorubicin; additionally, it reduced doxorubicin-induced organ toxicity and, therefore, has the potential to serve as a nutraceutical/complementary medicine.

Previously, cardiotoxic, nephrotoxic, and hepatotoxic effects of doxorubicin therapy [39,40,41] and cocoa in preventing multiple organ damage have also been reported [25,26,42]. COE treatment in combination with doxorubicin and pretreatment with the COE showed a significant decrease in the percentage change in body weight and ascites fluid volume in mice compared with the EAC group. Interestingly, we observed a significant decrease in the percentage change in body weight in the COE-pretreated mice compared with doxorubicin, suggesting the prophylactic activity of COE in cancer progression.

Previous, studies have reported that abnormal ECG patterns, such as increased width of the QRS complex, majorly contribute to ventricular hypertrophy, myocardial infarction, altered cardiac function, and other conduction abnormalities [43,44]. In the current study, an increase in the width of the QRS complex and a reduction in heart rate was observed in the EAC and doxorubicin groups. However, in all COE-treated groups, abnormal ECG resulting from doxorubicin and EAC was reversed, which suggests a cardioprotective role for COE against doxorubicin and EAC-induced cardiotoxicity.

Alterations in haematological parameters such as myelosuppression and anemia in the EAC mouse model have been well reported [45]. Similarly, in our present study, analogous findings were observed in EAC-bearing mice. In the treatment groups, both doxorubicin and COE reversed these haematological parameters in EAC-bearing mice; better amelioration was noted in COE group compared with the doxorubicin group, which suggests the beneficial effect of COE over doxorubicin treatment on haematopoiesis. In addition, it has been reported that anemia in EAC mice is caused by iron deficiency (haemolytic and myelopathic conditions), leading to a compromised RBC count [46]. In contrast, the RBC count in the doxorubicin and COE-treated mice was significantly increased compared with that in the EAC group, suggesting the beneficial role of COE along with doxorubicin treatment.

Elevated CPK-MB and LDH levels are considered important biomarkers of cardiac myocyte damage, especially during the clinical follow-up of doxorubicin therapy. The free radical generation during doxorubicin therapy causes considerable damage to the myocardium, which causes an increase in membrane permeability and thus the release of CPK-MB and LDH [47]. In the present study, doxorubicin treatment showed 2.54-fold and 5.41-fold increases in CPK-MB and LDH levels, respectively, compared with the normal group. COE treatment alone and in combination with doxorubicin significantly reduced the CPK-MB (2.10-fold and 1.16-fold, respectively) and LDH (3.67-fold and 3.53-fold, respectively) levels, reflecting the cardioprotective activity of COE. Along with these parameters, LPO elevated levels and GSH, SOD, CAT, and total thiol decreased levels in the EAC group were substantially reversed in all COE-treated groups compared with animals treated with doxorubicin alone. This is suggestive of COE scavenging free radicals generated during cancer propagation and doxorubicin therapy, thereby rendering beneficial effects to the host.

Nayagam (2019) [48] reported that doxorubicin-associated cardiotoxicity due to the accumulation of circulating free fatty acids (FFAs), leading to blockage of the coronary arteries. Cocoa ameliorates the lipid profile in dyslipidaemic conditions in the complex pathogenesis of lipid and glucose metabolism [49]. These two observations prompted us to assess the effect of COE on lipid-lowering properties in EAC and doxorubicin-induced hyperlipidaemia [50,51]. COE alone and in combination with doxorubicin significantly reversed the altered parameters, viz., HDL, cholesterol, and triglycerides, which reflected the shielding role of COE against hyperlipidaemia, which, in turn, prevented cardiac injury and preserved cardiac function.

Hepatocyte damage due to doxorubicin-induced ROS, specifically superoxide anions, compromises mitochondrial function and aggravates liver damage [52]. Similarly, EAC cells affect the liver through the accumulation of ascetic fluid and by the leakage of liver enzymes such as ALP, SGOT, and SGPT into serum [53]. Thus, the enzyme content in the liver serves as a biomarker for hepatotoxicity. In addition to the liver, doxorubicin and EAC also contribute to kidney damage. Previously, Mutar (2020) [54] reported that Ehrlich tumors as a reason for kidney damage by increasing urea and creatinine. These changes in urea and creatinine levels in the kidney contribute to the increased glomerular capillary permeability and tubular atrophy [55], which are responsible for kidney failure. In our study, the elevation of both hepatic and renal markers in the EAC and doxorubicin groups was observed, which was significantly reversed with COE treatment alone or in combination with doxorubicin. These findings support the beneficial role of COE against EAC- and doxorubicin-associated toxicities and demonstrate its vital role in regulating liver and kidney functions by balancing the serum biomarkers. Furthermore, a COE-mediated antioxidant defensive mechanism was also observed in both liver and kidney tissue. The elevated level of LPO and reduced levels of GSH, CAT, SOD, and thiols observed in both the EAC- and doxorubicin-treated groups were reversed in all COE-treated groups, establishing a beneficial role in cell protection from ROS and reducing organ toxicities concerning the heart, liver, and kidney.

Accumulation of fluid in the intravascular and interstitial spaces in cardiac tissue results in cardiac load, leading to congestion triggered by cardiac edema [56]. Mishra (2018) [57] demonstrated that EAC triggers cardiac dysfunction traced by congestion scores. In this study, a higher congestion score in EAC mice was observed compared with the treatment groups. Furthermore, edema was observed in the EAC- and doxorubicin-treated groups, which was reversed in all groups with COE treatment, indicating that COE potentially repaired the cardiac damage caused by doxorubicin and EAC.

Spotty necrosis, venous congestion, sinusoidal congestion, inflammation, and Kupffer cell hyperplasia serve as indicators of hepatic tissue damage [58,59]. In the present and previous studies, similar histopathological findings have also been identified in both EAC mice [60] and doxorubicin-treated animals [61]. However, in this study, animals treated with DOX alone did not show any improvement in spotty necrosis but showed reduced venous congestion, sinusoidal congestion, and inflammation. Furthermore, the COE in all treated groups showed significant improvement in the reduction of doxorubicin- and EAC-induced hepatic damage. This could be due to the accumulation of lipids and upregulated lipogenesis, as evidenced by upregulated LDL serum levels. The doxorubicin metabolite doxorubicinol is reported to upregulate the ROS system, which may disturb the homeostatic function of hepatocytes and alter multiple biological processes, cellular components, and molecular functions within it, leading to nonalcoholic fatty liver pathogenesis. These reports have been supported by our study. COE treatment was found to reverse EAC- and doxorubicin-induced liver damage. This could be due to potential antioxidant activity owing to the presence of certain biomarkers, such as catchin, (−)-epigallocatechin gallate (EGCG), hirsutrin, hyperoside, and cinaroside (molecules having higher hydrogen donating capacity).

Renal dysfunction induced by cardiac and hepatic injury leads to an increase in renal interstitial pressure on the entire capillary and tubules, which are triggered by an increase in glomerular and tubular congestion [62]. A similar renal dysfunction with elevated tubular and glomerular congestion has been reported in EAC- and doxorubicin-associatedtoxicities [63,64]. These effects could be due to the increase in the ROS system or free radicals and the presence of toxic doxorubicin metabolites in the nephron, which needs to be further investigated. In this study, similar histopathological observations were recorded in the EAC-induced and doxorubicin-treated animals. Interestingly, this damage was reversed in the COE-treated groups, suggesting the beneficial role of COE and nephroprotective activity during doxorubicin treatment in the carcinoma model.

In our study, the Ehrlich ascites model responded better to COE treatment compared with doxorubicin treatment. This may be due to the ascites microenvironment favouring oxidative stress [65], the proliferation of tumor cells [66], and comparatively less susceptibility of Ehrlich ascites cells towards oxidative stress [67]. These factors may also contribute to the reduced chemotherapeutic potential of doxorubicin in ascites tumor models compared with COE, due to its dual advantage of antioxidative and anticancer activities. In addition, COE also attenuate the adverse effects produced by doxorubicin on non-tumor cells without compromising its cancer therapeutic potential. This proves the protective effect of COE and suggesting it be considered as a health supplement during cancer chemotherapy.

## 5. Conclusions

The present study not only demonstrated the protective efficacy of COE against doxorubicin-induced organ (heart, liver, and kidney) toxicities in mice but also established its anticancer activity without compromising doxorubicin’s chemotherapeutic effect in the EAC model. Furthermore, our study demonstrated the ability of COE to neutralize the free radicals generated from doxorubicin and maintain cellular integrity along with its inherent anticancer properties to increase the survival time of EAC mice. Overall, COE was found to possess promising cardioprotective, hepatoprotective, and renoprotective activities when co-administered with doxorubicin. Further confirmatory studies at the clinical level are needed to establish cocoa as a nutraceutical against doxorubicin-induced organ damage.

## Figures and Tables

**Table 1:** Effect of COE on organ weight.

Groups	Heart (gms)	Liver (gms)	Kidney (gms)
Normal	0.115 ± 0.005	0.786 ± 0.097	0.296 ± 0.031
EAC	0.085 ± 0.002 ^▀^^ ^^▀^^ ^^▀^	2.033 ± 0.067 ^▀^^ ^^▀^^ ^^▀^	0.388 ± 0.014 ^▀^
DOX	0.136 ± 0.006 ^▲▲▲^	1.15 ± 0.050 ^▲▲▲^	0.418 ± 0.011
COE	0.116 ± 0.008 ^▲▲^	1.45 ± 0.067 ^▲▲▲^	0.348 ± 0.015
COE + DOX	0.121 ± 0.007 ^▲▲^	1.133 ± 0.033 ^▲▲▲^	0.385 ± 0.022
Pt + COE + DOX	0.113 ± 0.006 ^▲^	1.317 ± 0.125 ^▲▲▲^	0.336 ± 0.016

All values are expressed as the mean ± SEM (n = 10). Data were analysed using one-way analysis of variance (ANOVA) followed by Tukey’s test. ^▀^ *p* < 0.05, ^▀^^ ^^▀^^ ^^▀^ *p* < 0.001, compared with normal; ^▲^ *p* < 0.05, ^▲▲^ *p* < 0.01, ^▲▲▲^ *p* < 0.001, compared with EAC.

**Table 2:** Effect of COE on haematological parameters.

Groups	Normal	EAC	DOX	COE	COE + DOX	Pt + COE + DOX
RBC million (cell/cmm)	9.745 ± 0.719	5.640 ± 0.147 ^▀ ▀ ▀^	7.668 ± 0.117 ^▲▲^	7.487 ± 0.228 ^▲^	6.667 ± 0.397	7.240 ± 0.191 ^▲^
Hb (g %)	14.350 ± 0.851	7.867 ± 0.210 ^▀ ▀ ▀^	9.530 ± 0.387	11.370 ± 0.705 ^▲▲▲^	10.320 ± 0.234 ^▲^	11.030 ± 0.255 ^▲▲^
Platelet count (cell/cmm)	14.850 ± 2.373	9.670 ± 0.609	17.58 ± 1.840 ^▲▲^	11.380 ± 0.368	12.850 ± 0.699	14.460 ± 0.795
PCV (%)	63.330 ± 1.553	38.750 ± 1.790 ^▀ ▀ ▀^	31.980 ± 1.677 ^▲^	35.580 ± 1.864	34.250 ± 1.404	37.180 ± 0.700
MCV (fl)	60.720 ± 2.585	53.680 ± 0.743	50.970 ± 1.861	51.000 ± 1.022	50.830 ± 0.784	52.020 ± 2.161
MCHC (gm/dL)	25.070 ± 0.507	28.480 ± 0.231 ^▀ ▀ ▀^	30.370 ± 0.364 ^▲▲^	30.870 ± 0.269 ^▲▲▲^	30.570 ± 0.172 ^▲▲▲^	30.880 ± 0.188 ^▲▲▲^
WBCs (cell/cmm)	19.810 ± 3.502	63.320 ± 10.0 ^▀ ▀ ▀^	20.5000 ± 2.997 ^▲▲▲^	25.710 ± 3.587 ^▲▲▲^	20.950 ± 2.527 ^▲▲▲^	21.620 ± 1.410 ^▲▲▲^
Lymphocytes (%)	88.000 ± 1.238	82.000 ± 3.406	83.500 ± 3.575	83.000 ± 4.619	73.000 ± 0.966	68.330 ± 0.882 ^▲^^●●^
Neutrophils (%)	7.833 ± 0.8333	15.670 ± 1.406 ^▀ ▀^	10.500 ± 1.893	7.333 ± 0.919 ^▲▲^	12.170 ± 0.872	13.670 ± 2.028
Eosinophils (%)	1.167 ± 0.4014	2.000 ± 1.000	1.667 ± 0.333	1.333 ± 0.210	1.000 ± 0.2582	1.000 ± 0.2582
Monocytes (%)	1.500 ± 0.223	5.000 ± 1.342	1.000 ± 0.000 ^▲▲^	2.500 ± 0.562	2.167 ± 0.477 ^▲^	1.667 ± 0.333 ^▲^
MCH (pg)	15.280 ± 0.393	14.250 ± 0.081	16.15 ± 0.474 ^▲▲^	15.58 ± 0.339	15.680 ± 0.119 ^▲^	15.430 ± 0.360

All values are expressed as the mean ± SEM (n = 6). Data were analysed using one-way analysis of variance (ANOVA) followed by Tukey’s test. ^▀^^ ^^▀^*p* < 0.01, ^▀^^ ^^▀^^ ^^▀^ *p* < 0.001, compared with normal; ^▲^ *p* < 0.05, ^▲▲^ *p* < 0.01, ^▲▲▲^ *p* < 0.001, compared with EAC; ^●●^ *p* < 0.01, compared with DOX.

**Table 3:** Effect of COE on CPK-MB and LDH.

Groups	CPK-MB (U/L)	LDH (U/L)
Normal	183.8 ± 9.940	4933 ± 1573.000
EAC	380.2 ± 64.18 ^▀^	17,843 ± 1718.000 ^▀ ▀ ▀^
DOX	467.2 ± 36.500	26,717 ± 1301.000 ^▲▲▲^
COE	223.3 ± 8.780 ^▲^	7125 ± 696.300 ^▲▲▲^
COE + DOX	402.5 ± 22.030	7550 ± 874.700 ^▲▲▲^^●●●^
Pt + COE + DOX	386.0 ± 25.430	6910 ± 600.600 ^▲▲▲^^●●●^

All values are expressed as the mean ± SEM (n = 6). Data were analysed using one-way analysis of variance (ANOVA) followed by Tukey’s test. ^▀^ *p* < 0.05, ^▀^^ ^^▀^^ ^^▀^ *p* < 0.001, compared with normal; ^▲^ *p* < 0.05, ^▲▲▲^ *p* < 0.001, compared with EAC; ^●●●^ *p* < 0.001, compared with DOX.

**Table 4:** Effect of COE on hepatic enzymes.

Groups	ALP (U/L)	SGOT (U/L)	SGPT (U/L)
Normal	20.83 ± 0.401	201 ± 4.690	75.5 ± 2.291
EAC	82.17 ± 4.622 ^▀ ▀ ▀^	1196 ± 10.110 ^▀ ▀ ▀^	264 ± 1.915 ^▀ ▀ ▀^
DOX	31.17 ± 1.447 ^▲▲▲^	1070 ± 46.560 ^▲^	184.8 ± 2.12 ^▲▲▲^
COE	22.33 ± 0.557 ^▲▲▲^	553.8 ± 22.530 ^▲▲▲^	110.3 ± 0.802 ^▲▲▲^
COE + DOX	27.5 ± 1.784 ^▲▲▲^	885.8 ± 13.670 ^▲▲▲^^●●●^	134.2 ± 1.276 ^▲▲▲^^●●●^
Pt + COE + DOX	27.67 ± 1.054 ^▲▲▲^	814.2 ± 27.260 ^▲▲▲^^●●●^	118.8 ± 1.493 ^▲▲▲^^●●●^

All values are expressed as the mean ± SEM (n = 6). Data were analysed using one-way analysis of variance (ANOVA) followed by Tukey’s test. ^▀^^ ^^▀^^ ^^▀^ *p* < 0.001, compared with normal; ^▲^ *p* < 0.05, ^▲▲▲^ *p* < 0.001, compared with EAC; ^●●●^ *p* < 0.001, compared with DOX.

**Table 5:** Effect of COE on creatinine and BUN.

Groups	Creatinine (mgs %)	BUN (mgs %)
Normal	0.125 ± 0.010	30.83 ± 0.980
EAC	0.333 ± 0.020 ^▀ ▀ ▀^	79.83 ± 0.477 ^▀ ▀ ▀^
DOX	0.233 ± 0.020 ^▲▲▲^	53.17 ± 0.654 ^▲▲▲^
COE	0.183 ± 0.010 ^▲▲▲^	34.50± 1.147 ^▲▲▲^
COE + DOX	0.200 ± 0.000 ^▲▲▲^	49.33 ± 0.333 ^▲▲▲^^●^
Pt + COE + DOX	0.191 ± 0.008 ^▲▲▲^	39.00 ± 0.577 ^▲▲▲^^●●●^

All values are expressed as the mean ± SEM (n = 6). Data were analysed using one-way analysis of variance (ANOVA) followed by Tukey’s test. ^▀^^ ^^▀^^ ^^▀^ *p* < 0.001, compared with normal; ^▲▲▲^ *p* < 0.001, compared with EAC; ^●^ *p* < 0.05, ^●●●^ *p* < 0.001, compared with DOX.

**Table 6:** Effect of COE on lipid profile.

Groups	Normal	EAC	DOX	COE	COE + DOX	Pt + COE + DOX
HDL(mgs %)	62.85 ± 6.419	24.00 ± 1.949 ^▀ ▀ ▀^	23.33 ± 1.202	43.50 ± 0.806 ^▲▲^	30.50 ± 0.670	33.50 ± 0.763
LDL (mgs %)	42.33 ± 1.229	48.67 ± 2.552	36.83 ± 1.014 ^▲▲^	38.67 ± 2.704 ^▲^	38.17 ± 2.587 ^▲^	39.67 ± 2.39
Cholesterol (mgs %)	84.83 ± 2.088	94.50 ± 4.272	115.0 ± 2.582 ^▲▲^	70.00 ± 2.033 ^▲▲▲^	89.83 ± 5.747 ^●●●^	88.17 ± 3.60 ^●●●^
Triglycerides (mgs %)	42.50 ± 0.885	74.00 ± 7.870 ^▀ ▀^	75.50 ± 11.690	23.50 ± 2.630 ^▲▲▲^	72.33 ± 2.603	69.50 ± 4.653

All values are expressed as the mean ± SEM (n = 6). Data were analysed using one-way analysis of variance (ANOVA) followed by Tukey’s test. ^▀^^ ^^▀^ *p* < 0.01, ^▀^^ ^^▀^^ ^^▀^ *p* < 0.001, compared with normal; ^▲^ *p* < 0.05, ^▲▲^ *p* < 0.01, ^▲▲▲^ *p* < 0.001, compared with EAC; ^●●●^ *p* < 0.001, compared with DOX.

**Table 7:** Effect of COE on enzymatic and nonenzymatic antioxidant biomarkers in heart homogenate.

Groups	LPO (Nano Moles/mg of Protein)	GSH (µMol/mg Protein)	SOD (Units/mg of Protein)	CAT (Units/mg of Protein)	Total Thiol (µMol/mg of Protein)
Normal	196.000 ± 19.100	18.050 ± 2.400	462.500 ± 12.000	0.790 ± 0.600	22.260 ± 3.200
EAC	508.000 ± 17.600 ^▀ ▀ ▀^	13.150 ± 0.200 ^▀ ▀ ▀^	182.600 ± 13.200 ^▀ ▀ ▀^	0.060 ± 0.001 ^▀ ▀ ▀^	11.430 ± 0.400 ^▀ ▀^
DOX	218.200 ± 18.500 ^▲▲▲^	15.070 ± 0.800	306.100 ± 15.900 ^▲▲^	0.450 ± 0.020 ^▲▲▲^	29.220 ± 1.400 ^▲▲▲^
COE	215.400 ± 8.100 ^▲▲▲^	24.990 ± 2.100 ^▲▲▲^	521.200 ± 14.300 ^▲▲▲^	0.650 ± 0.070 ^▲▲▲^	38.440 ± 2.700 ^▲▲▲^
COE + DOX	148.500 ± 7.700 ^▲▲▲^^●^	20.260 ± 0.500 ^▲▲^	436.100 ± 23.900 ^▲▲▲^^●●^	0.560 ± 0.010 ^▲▲▲^	35.150 ± 0.900 ^▲▲▲^^●^
Pt + COE + DOX	149.900 ± 11.200 ^▲▲▲^^●^	22.020 ± 1.000 ^▲▲▲^^●^	413.700 ± 34.700 ^▲▲▲^^●^	0.550 ± 0.050 ^▲▲▲^	38.700 ± 0.800 ^▲▲▲^^●^

All values are expressed as the mean ± SEM (n = 6). Data were analysed using one-way analysis of variance (ANOVA) followed by Tukey’s test. ^▀^^ ^^▀^ *p* < 0.01, ^▀^^ ^^▀^^ ^^▀^ *p* < 0.001, compared with normal; ^▲▲^ *p* < 0.01, ^▲▲▲^ *p* < 0.001, compared with EAC; ^●^ *p* < 0.05, ^●●^ *p* < 0.01 compared with DOX.

**Table 8:** Effect of COE on enzymatic and nonenzymatic antioxidant biomarkers in the liver homogenate.

Groups	LPO (Nano Moles/mgof Protein)	GSH (µMol/mg Protein)	SOD (Units/mg of Protein)	CAT (Units/mg of Protein)	Total Thiol (µMol/mg of Protein)
Normal	193.300 ± 14.300	32.500 ± 0.600	427.500 ± 16.300	0.980 ± 0.180	142.100 ± 6.700
EAC	649.000 ± 57.300 ^▀^	10.070 ± 0.300 ^▀^	156.900 ± 19.200 ^▀^	0.140 ± 0.050 ^▀ ▀^	19.670 ± 1.80 ^▀^
DOX	368.800 ± 49.900 ^▲▲▲^	22.470 ± 1.100	389.40 ± 21.200 ^▲▲▲^	1.270 ± 0.150 ^▲▲▲^	37.750 ± 5.700 ^▲^
COE	364.700 ± 32.500 ^▲▲▲^	39.800 ± 1.700 ^▲▲▲^	595.800 ± 27.900 ^▲▲▲^	1.200 ± 0.150 ^▲▲^	115.300 ± 3.700 ^▲▲▲^
COE + DOX	308.300 ± 16.500 ^▲▲▲^	33.510 ± 2.400 ^▲▲▲^	428.300 ± 07.500 ^▲▲▲^	0.900 ± 0.190 ^▲▲▲^	95.220 ± 1.300 ^▲▲▲^^●●●^
Pt + COE + DOX	210.000 ± 7.200 ^▲▲▲^^●^	36.490 ± 1.500 ^▲▲▲^	495.700 ± 26.500 ^▲▲▲^^●^	1.320 ± 0.120 ^▲▲▲^	77.410 ± 2.200 ^▲▲▲^^●●^

All values are expressed as the mean ± SEM (n = 6). Data were analysed using one-way analysis of variance (ANOVA) followed by Tukey’s test. ^▀^ *p* < 0.05, ^▀^^ ^^▀^ *p* < 0.01, compared with normal; ^▲^ *p* < 0.05, ^▲▲^ *p* < 0.01, ^▲▲▲^ *p* < 0.001, compared with EAC; ^●^ *p* < 0.05, ^●●^ *p* < 0.01, ^●●●^ *p* < 0.001, compared with DOX.

**Table 9:** Effect of COE on enzymatic and non-enzymatic antioxidant biomarkers in kidney homogenate.

Groups	LPO (nM/mg of Protein)	GSH (µM/mg Protein)	SOD (U/mg of Protein)	CAT (U/mg of Protein)	Total Thiol (µM/mg of Protein)
Normal	214.200 ± 31.100	21.600 ± 0.300	496.600 ± 64.600	0.750 ± 0.120	68.850 ± 10.600
EAC	439.300 ± 24.800 ^▀ ▀ ▀^	12.290 ± 0.300 ^▀ ▀ ▀^	182.500 ± 5.700 ^▀ ▀ ▀^	0.140 ± 0.050 ^▀ ▀ ▀^	25.450 ± 2000 ^▀ ▀ ▀^
DOX	227.300 ± 9.704 ^▲▲▲^	12.280 ± 0.400	314.900 ± 26.000	0.470 ± 0.060 ^▲^	30.490 ± 2.500
COE	207.900 ± 8.100 ^▲▲▲^	23.370 ± 0.700 ^▲▲▲^	490.700 ± 23.600 ^▲▲▲^	0.570 ± 0.070 ^▲▲^	63.190 ± 5.00 ^▲▲^
COE + DOX	213.800 ± 17.100 ^▲▲▲^	21.380 ± 0.200 ^▲▲▲^^●●●^	364.700 ± 31.200 ^▲^	0.490 ± 0.060 ^▲^	44.190 ± 3.900
Pt + COE + DOX	222.900 ± 17.700 ^▲▲▲^	22.690 ± 0.600 ^▲▲▲^^●●●^	430.200 ± 46.800 ^▲▲^	0.530 ± 0.050 ^▲^	55.170 ± 6.300 ^▲^

All values are expressed as the mean ± SEM (n = 6). Data were analysed using one-way analysis of variance (ANOVA) followed by Tukey’s test. ^▀^^ ^^▀^^ ^^▀^ *p* < 0.001, compared with normal; ^▲^ *p* < 0.05, ^▲▲^ *p* < 0.01, ^▲▲▲^ *p* < 0.001, compared with EAC; ^●●●^ *p* < 0.001, compared with DOX.

## Data Availability

The original contributions presented in the study are included in the article, further inquiries can be directed to the corresponding author.

## Abbreviations

ALP: Alkaline phosphatase; BUN: Blood urea nitrogen; CAT: Catalase; COE: hydroalcoholic extract of cocoa bean; CPK-MB: Creatine phosphokinase-MB; DOX: Doxorubicin-treated animal group; EAC: Ehrlich ascites carcinoma; EDTA: Ethylenediaminetetraacetic acid; GSH: Glutathione; HDL: High-density lipoprotein; Hb: Haemoglobin; ILS: Increased life span; LDL: Low-density lipoproteins; LDH: Lactate dehydrogenase; LPO: Lipid peroxidation; MCH: Mean corpuscular haemoglobin; MCHC: Mean corpuscular haemoglobin concentration; MST: Mean survival time; PCV: Packed Cell Volume; RBC: Red blood cells; ROS: Reactive oxygen species; SGOT: Serum glutamic-oxaloacetic transaminase; SGPT: Serum glutamic pyruvic transaminase; SOD: Superoxide dismutase; WBC: White blood cells.
